# Construction and Validation of the HeiQ: An Operation-Oriented Figural Matrices Test

**DOI:** 10.3390/jintelligence11040073

**Published:** 2023-04-18

**Authors:** Vanessa S. Pallentin, Daniel Danner, Jan Rummel

**Affiliations:** 1 Department of Psychology, Heidelberg University, Hauptstr. 47-51, 69117 Heidelberg, Germany; 2 Section Psychology, University of Applied Labour Studies, Seckenheimer Landstr. 16, 68163 Mannheim, Germany

**Keywords:** figural matrices test, intelligence, reasoning, Rasch analysis

## Abstract

Figural matrices tests are among the most popular and well-investigated tests used to assess inductive reasoning abilities. Solving these tests requires the selection of a target that completes a figural matrix among distractors. Despite their generally good psychometric properties, previous matrices tests have limitations associated with distractor construction that prevent them from realizing their full potential. Most tests allow participants to identify the correct response by eliminating distractors based on superficial features. The goal of this study was to develop a novel figural matrices test which is less prone to the use of response elimination strategies, and to test its psychometric properties. The new test consists of 48 items and was validated with *N* = 767 participants. Measurement models implied that the test is Rasch scalable, inferring a uniform underlying ability. The test showed good to very good reliability (retest-correlation: *r* = 0.88; Cronbach’s alpha: *α* = 0.93; split-half reliability: *r* = 0.88) and good construct validity (*r* = 0.81 with the Raven Progressive Matrices Test, *r* = 0.73 with global intelligence scores of the Intelligence Structure Test 2000R, and *r* = 0.58 with the global score of the Berlin Intelligence Structure Test). It even superseded the Raven Progressive Matrices Tests in criterion-related validity (correlation with final year high school grades (*r* = −0.49 *p* < .001)). We conclude that this novel test has excellent psychometric properties and can be a valuable tool for researchers interested in reasoning assessment.

## 1. Introduction

Fluid reasoning abilities, which are sometimes also called fluid intelligence, are one of the best predictors of educational achievement (Deary et al. 2007), job performance (Schmidt and Hunter 2004), health and longevity (Gottfredson and Deary 2004), and complex problem solving (Danner et al. 2011). Within the Cattell–Horn–Caroll taxonomy of cognitive abilities, fluid reasoning is defined as the ability to identify underlying rules or patterns in novel situations (Cattell 1963; Horn 1968; Horn and Cattell 1967; McGrew 2009). Thus, people with good fluid reasoning abilities are good at solving inductive reasoning problems without having the prior knowledge, scripts, habits, or schemata to do so (Schneider and McGrew 2012). For this reason, fluid reasoning abilities are typically assessed using figural reasoning tests, such as the prominent Raven matrices test (Carpenter et al. 1990; Marshalek et al. 1983; Raven et al. 1994; Waschl et al. 2016). Although several figural reasoning tests have been developed and their validity has been established, we believe that there is room for improvement in the assessment of inductive reasoning abilities. For this purpose, we developed a new test to overcome the psychometric weaknesses of the currently available tests.

**Figure 1:**
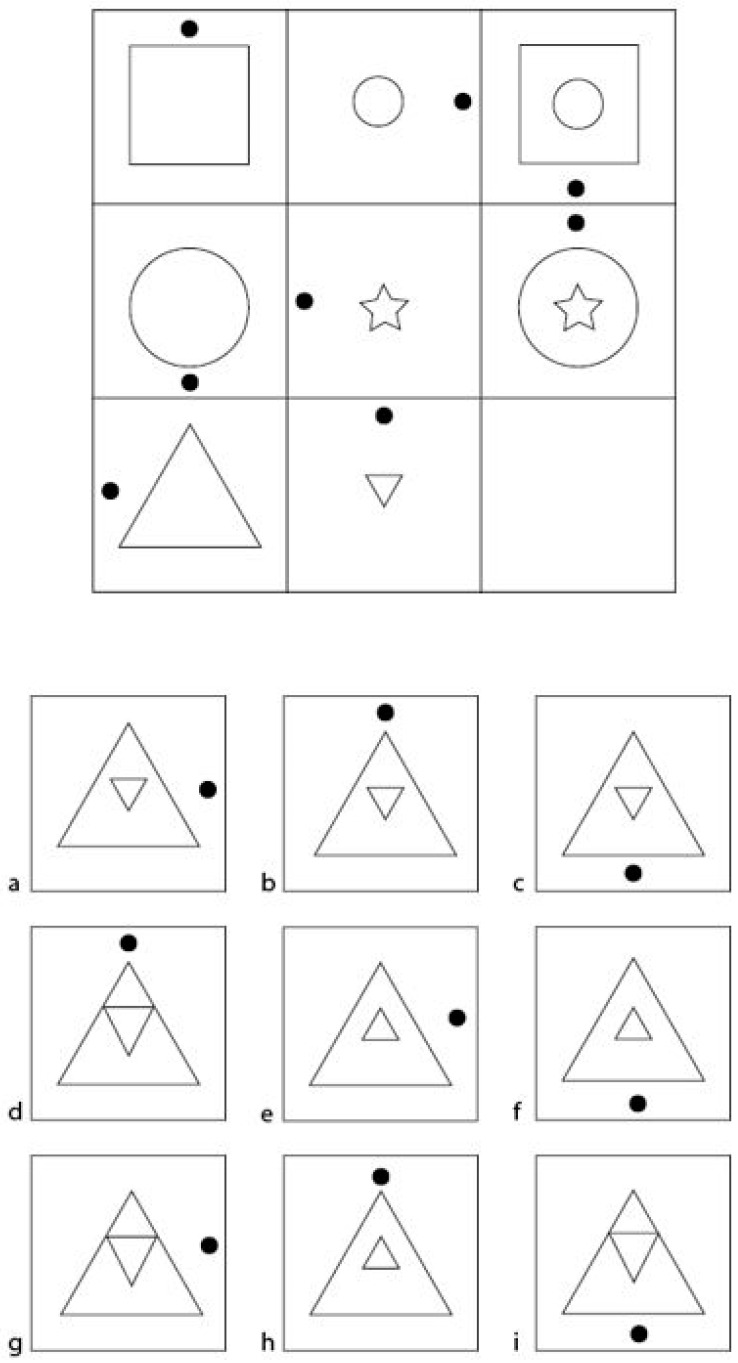
Illustration of a typical (easy) matrices test item. This test item is taken from the practice item section of our newly developed figural matrices test. In this example item, response option a is correct and options b to i are incorrect. Both operations used to construct the figures are vertically applied. The two central elements of the two figures on the left and in the middle are combined to form the third figure in each matrix row, while the black dot rotates clockwise in steps of 90 degrees from left to right.

Items of figural reasoning tests typically consist of a matrix, each cell of which shows a figure representing a combination of different elements. From cell to cell, the figures’ elements may vary in shape, texture, color, number, orientation, and/or arrangement (Formann 1973). The figures are assigned to matrix cells following certain operations, which may be applied column-wise, row-wise, or both (Formann 1973). Critically, one cell is always left empty, and the participants’ task is to identify the single figure that will complete the matrix, the so-called attractor, in accordance with the underlying operations among a set of candidate figures consisting of the attractor and several distractors. An exemplary matrices test item is displayed in Figure 1.

Items of earlier tests, such as the Raven Matrices Test (Raven 1976) or the Wiener Matrices Test (Formann and Piswanger 1979) were developed in an intuitive fashion, meaning that the items were created based on the personal intuition of the test constructors and then tested on samples to decide which items to keep (Hornke et al. 2000; Klauer 1978; Mittring and Rost 2008; Schott and Wieberg 1984). Such intuitive approaches have been criticized for being created by “testing specialists who based their advice on personal experience, wisdom and limited empirical research” (Haladyna et al. 2002, p. 310). More recently, item construction has been approached in a more rational manner, meaning that test items are created by following explicit construction guidelines (Haladyna and Downing 1989; Heydasch 2014; Hornke et al. 2000; Hornke and Habon 1986). Typical examples for matrices tests developed under a rational approach are the Hagen Matrices Test (Heydasch 2014) and the Bochumer Matrices Test (Hossiep et al. 1999). The Hagen Matrices Test also uses a 3 × 3 matrix, whilst the Bochumer Matrices Test items are arranged in a 5 × 3 matrix. They are both based on a construction framework with pre-defined rules for item generation, which is also why they are also labelled ‘rule-based’. In the given context, the term rule refers to the mental operations that participants need to detect and solve. Therefore, in the remainder of this article, we will refer to these mental operations simply as operations, in line with the nomenclature of Hornke and Habon (1986). Figural reasoning test items can also be generated in an automated fashion by using computer applications such as the MatrixDeveloper (Freund et al. 2008) or the International Cognitive Ability Resource ICAR (The International Cognitive Ability Resource Team 2014). The MatrixDeveloper features six different operations from which the user can choose for generating items (Jendryczko et al. 2019). The tool then generates the item accordingly, randomly selecting shapes, color (black or white), or symbols (Jendryczko et al. 2019).

Notably, even among the tests that used a rational approach, there is currently no royal road to test the construction of figural reasoning tests items. The construction of distractors, especially, has not received much attention to date. The few yet proposed design strategies only relate to some rather specific construction aspects, and most of them have not been validated (Becker et al. 2014). Even with the automated item generators, the distractors are not constructed in a fully systematized fashion. For this reason, we established a new rationale for a systematized distractor generation for our new test with the aim of achieving optimal psychometric properties.

When solving matrices tests, participants regularly use a constructive matching strategy, that is, they identify and apply the underlying operations, mentally construe the answer, and then choose the response option that best reflects the imagined solution (Gonthier and Roulin 2020). For the example in Figure 1, participants would look at the upper 3 × 3 matrix, the item stem, and draw a mental picture of the solution. Hence, the black dot needs to be on the right-hand side, and the inversed smaller triangle needs to be within the bigger triangle with the proportions staying equal. With this mental picture, they would look at the response options and choose the matching option, which is response option a. Sometimes, however, participants can also rely on a bypassing strategy called response elimination (Bethell-Fox et al. 1984; Hayes et al. 2011). Rather than identifying the operations underlying an item, participants inspect the candidate figures to exclude distractors (Arendasy and Sommer 2013; Becker et al. 2016). By doing so, implausible distractors that do not follow any of the operations used to construct the item can be easily eliminated (Case and Swanson 2002; Gierl et al. 2017; Haladyna and Downing 1989; La Torre and Karelitz 2009). For example, if an additional figural element is added to every cell in the third column from left to right, as it is the case in Figure 1, a distractor featuring fewer elements than the cells in the middle column can easily be dismissed. In the same vein, distractors that are visually dissimilar to all other response options can be eliminated without applying any operation (Arendasy and Sommer 2013; Jarosz and Wiley 2012). For instance, if only one distractor features a circle and all other response options solely feature triangles and squares, participants are able to directly rule out this single distractor. For the example in Figure 1, a distractor that would feature any shape other than a triangle for the inner or outer figural element would easily be identified as such.

In a worst-case scenario, participants can use a response elimination strategy to identify the correct response without considering the matrix at all (Mittring and Rost 2008). Usually, any single distractor will feature more figural elements that are also a part of the solution than elements that are not a part of the solution; however, each distractor will feature a different subset of elements from the solution. By mere inspection of all candidate figures, participants can identify the one that shows the highest number of recurring elements and, in doing so, identify the attractor without having any insight into the underlying operations. This bypassing strategy has been referred to as counting (Mittring and Rost 2008). Mittring and Rost (2008) showed that for approximately 50% of the items of the Raven Advanced Progressive Matrices (RAPM) (Raven 1976), the attractor can be unanimously identified by applying such a bypassing strategy without having seen the test matrices of these items. More recently developed matrices tests, including those that employed a rational item construction approach, do not perform much better in this regard: for the Bochum Matrices Test Advanced (Hossiep et al. 1999), 35% of items can be solved by bypassing strategies; for the Naglieri Nonverbal Ability Test (Naglieri 2003) it is 70%; for the test for intellectual giftedness (Preckel 2003) it is 60%; and for the Wiener Matrizen Test (Formann and Piswanger 1979) it is 75% of the items.

These percentages are significant in that they reflect the proportion of items for which the correct response was unequivocally identified. Therefore, even if an item cannot be solved by using bypassing strategies, the likelihood of choosing the correct response option increases when participants can dismiss certain distractors right away (Arendasy and Sommer 2013; Becker et al. 2016; Tarrant et al. 2009). For the test for intellectual giftedness (Preckel 2003), Mittring and Rost (2008) report that for an additional 15% of the test items, the guessing probability can be increased to *p* = .50, if the bypassing strategy of counting is used.

Participants tend to use bypassing strategies if possible because they are less cognitively effortful than constructive matching strategies (Gonthier and Roulin 2020). The downside of such bypassing attempts is that test performance typically suffers from them (Arendasy and Sommer 2013; Bethell-Fox et al. 1984; Gonthier and Roulin 2020). Although participants with better cognitive abilities are generally less likely to use bypassing strategies than participants with lower abilities, the reliance on such strategies will influence test performance beyond cognitive abilities (Gonthier and Roulin 2020). It has been shown, for example, that a participant with a lower working memory capacity who uses a constructive matching strategy can outperform a participant with a higher capacity who uses response elimination as a bypassing strategy (Gonthier and Roulin 2020). The scores of participants who used a bypassing strategy can be thus expected to be less indicative of their actual ability than the scores of participants who did not. Taken together, when participants extensively use bypassing strategies for solving a matrices test, the convergent validity with other cognitive abilities tests is likely to be reduced (Arendasy and Sommer 2013; Becker et al. 2016).

In light of these observations, we aimed to develop a novel matrices test that allows for a more precise assessment of reasoning abilities and limits and prevents participants from relying on response elimination strategies, especially counting. To our best knowledge, there are no clear construction guidelines to prevent response elimination and counting strategies. However, the work by Guttman and Schlesinger (1967) is useful in this regard, as they proposed a general systematic distractor construction approach for ability and achievement tests. According to this approach, if the test content is a priori explicated in a facet design, distractors can be generated so that they systematically follow the same design. Within the facet design framework, if a figural reasoning test features a finite number of operations, each distractor needs to portray a different combination of correctly and incorrectly applied operations, meaning distractors need to be collectively exhaustive (Guttman and Schlesinger 1967). For example, if an item consists of two operations, operations A and B, the same amount of distractors out of the total amount of distractors must correctly (1) display operation A, but not operation B, (2) correctly display operation B, but not operation A, and (3) incorrectly display operation A and operation B. Bypassing strategies make use of the fact that certain or even all distractors can be dismissed based on some visual and structural features (Arendasy and Sommer 2013). A direct consequence of the systematic distractor generation approach, however, will be that distractors will be structurally similar to the attractor, which will render them the most plausible.

Furthermore, if the operations used to construct an item are made explicit, the mental operations a participant relies on to solve a particular item can be deduced. In as much as these operations are reflective of the mental processes underlying the latent ability assessed with the item, the analysis of operation detection can be indicative of the cognitive processes at work during item solution (Hornke and Habon 1986; Undeutsch 2010). As responses to items are typically simply scored as “correct versus incorrect”, information about the participants’ reasoning that led to the selection of a particular distractor is lost (Laurence and Macedo 2022). The importance of analyzing distractors for their informative content has come into focus in recent years (Forthmann et al. 2020; Storme et al. 2019). While the focus of previous work was dedicated to analyzing single distractors for ability, our approach focuses on increasing distractor information by using the interplay of distractors and their operations resulting from the facet design (Guttman and Schlesinger 1967). Under the systematic distractor generation approach, combinations of correctly and incorrectly applied operations within each distractor can be used to infer which operations were understood and applied correctly by a participant who chose a particular distractor (Guttman and Schlesinger 1967). Simply put, distractor analysis can help us to better understand the cognitive processes at work in a given person while solving a particular item (Forthmann et al. 2020; Laurence and Macedo 2022).

Based on the considerations just outlined, for our novel matrices test we developed and used a systematic distractor generation approach with the following features:

- Distractors and the attractor were generated so that the attractor cannot be identified by using a counting strategy (Mittring and Rost 2008);
- Distractors and the attractor were generated so that they are of a similar visual appearance (Arendasy and Sommer 2013);
- All distractors were generated so that they were plausible solutions to the item (Gierl et al. 2017; Haladyna et al. 2002; Haladyna and Rodriguez 2013): plausibility was achieved by applying some, but not all of the item’s underlying operations correctly when generating a distractor (Guttman and Schlesinger 1967) and by applying additional operation(s) in accordance with possible misconceptions about the operations used in the respective item (Case and Swanson 2002);
- Each possible correct/incorrect combination of the item’s underlying operations was reflected by one particular distractor, so that distractor selection would be informative with regard to which operations were correctly applied and which were incorrectly applied (Guttman and Schlesinger 1967).

For the novel matrices test, we intended to generate items that cover a wide range of abilities and would thus allow us to measure and compare reasoning abilities across different populations. For this purpose, we intended to generate items which were more and items which were less easy to solve. We further intended to apply Rasch model scaling to the data so that item difficulties and a person’s abilities could be estimated independently. The Rasch model implies that all items load equally strongly on the latent ability they are assumed to assess, thus allowing the use of a sum score and supporting the unidimensionality of the underlying ability (Rasch 1960). In order to generate the required number of distractors per item under our approach, every item must feature at least two operations. For our test, we decided to come up with items consisting of two as well as items consisting of three operations. Items usually become more difficult when the number and complexity of their underlying operations increase (Carpenter et al. 1990; Gonthier and Roulin 2020). This is because before participants can apply the specific operations they have to distinguish the figural elements within a matrix that reflect the different operations and correspond to each other in order to identify them (Carpenter et al. 1990). Furthermore, if participants succeed in detecting and solving one operation underlying an item that features two or more operations, they have to hold this information in their memory, while trying to identify and solve the other operations (Carpenter et al. 1990). Because either two or three operations from the same finite set of operations underlie all the items of our new test, we are better able to determine item difficulties (Freund et al. 2008). Items with three underlying operations should be more difficult than items with two. Additionally, because distractors partially follow the same operations as the attractors, we should be able to more precisely determine the difficulties of distractors based on their similarity with the attractor (Guttman and Schlesinger 1967).

## 2. Materials and Methods

### 2.1. Participants

For the present study, data from 5 different sub-samples, all collected between March 2020 and December 2021, were pooled. In total, 3 sub-samples consisted of student participants; the other 2 consisted of participants from the general population. Participants were recruited in lectures, from the university participant pool, through a newspaper article, by e-mail, and through posts to Facebook groups. Data were collected partly online and partly in the laboratory. All participants filled in the HeiQ and a subset of other tests and questionnaires depending on the sub-sample they belonged to (see below). A total of *N* = 733 participants completed the study.

Participants who reported that they did not follow the instructions by confirming item “I often clicked something just to finish the study quickly” presented at the very end of the study were excluded (*N* = 13). Sub-samples 2 to 5 were presented with a catch item that was easily solvable but looked like a regular item on first sight. Participants who did not solve this item were excluded from further analysis (*N* = 6). Online participants (from sub-samples 1, 2, and 3) were not given a time limit, but had been instructed to perform the test without interruptions. Participants who took more than 4 h to answer all test items were excluded (*N* = 13) after an outlier analysis. Given the median test duration of 57.52 min for the remaining participants of the 3 sub-samples (*N* = 388), such long test-taking time was considered an indication of a longer interruption during test completion. Some participants completed the matrices test in less than 2 min, which indicates that they did not try to solve it properly. To exclude items that were not seriously attended to, item solution attempts that took less than 5 s were considered as overly fast and coded as missing values. A 5 s cut-off was chosen because, in our sample, the single fastest correct solution, which was provided by 1 participant who solved all of the items correctly, took 6 s. Furthermore, in the previous research on fast responses it has been argued that participants require at least 5 s to examine, understand, and answer an item (Wise 2017). Participants who showed overly fast responses on 50% of all test items in the HeiQ were completely excluded because they likely did not take the test seriously (*N* = 18).

The final sample consisted of *N* = 683 participants. These participants’ mean age was *M* = 25.88 (*SD* = 9.04); 66.2% were female, 32.7% were male, and 0.3% identified as non-binary or preferred not to say. A total of 98.4% resided in Germany, with the remainder residing in Europe. Further, *N* = 590 (86.4%) participants’ first language was German, *N* = 20 (2.9%) reported they were raised bilingual with German being 1 of the 2 mother languages, and the remaining participants reported their first language was not German or preferred not to say. Most participants were university students (84.3%). Further, *N* = 467 participants and performed the study online; *N* = 216 participants performed it in the laboratory. Demographic information for each sub-sample and an overview of the constructs assessed within each sub-sample are provided in Table 1.

### 2.2. Materials

#### 2.2.1. Construction of the Heidelberg Figural Matrices Test (HeiQ)

The new test introduced in this study was the figural matrices test HeiQ (pronounced “high-q”). The test consisted of 48 items plus one catch item administered for four of the five sub-samples. Each item consisted of a 3 × 3 matrix, of which the very right field in the lowest row is empty. Participants had to identify the correct response option (the attractor) which completes the pattern within the matrix. The attractor was presented together with seven distractors for items with two operations or eight distractors for items with three operations below the matrix on the screen. The position of the attractor among the distractors varied randomly from item to item but was fixed across participants.

##### Operations

**Figure 2:**
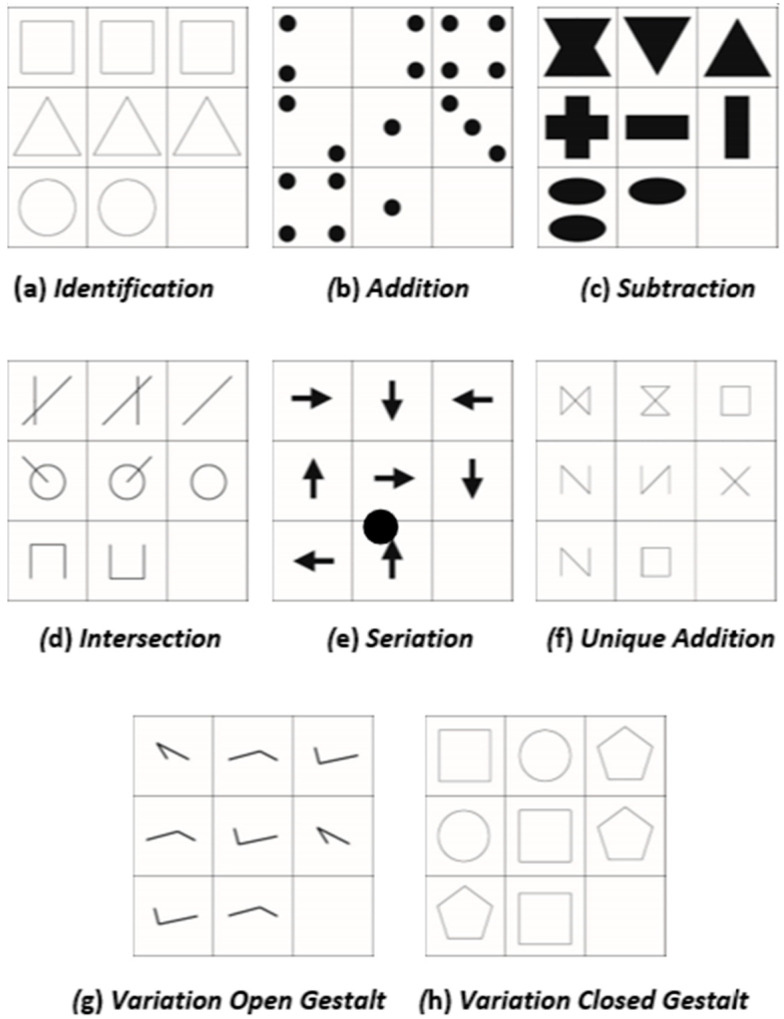
Overview of the eight operations that are used to construct the items. See (Hornke and Habon 1986) for a similar example.

Each cell of the 3 × 3 matrix contains several geometric shapes (e.g., squares; triangles), lines, and/or patterns, which change from cell to cell following certain regularities. For each item, we used two or three out of eight possible operations (Hornke et al. 2000; Hornke and Habon 1986). Figure 2 illustrates the different operations we used. The operations are:

- Identity (ID): The same figure is repeatedly displayed in each cell of a row/column, (Figure 2a);
- Addition (AD): The figures of the first two cells (row-/column-wise) are added together to form the third figure (Figure 2b);
- Subtraction (SU): The figure of the second cells (row-/column-wise) is deducted from the figure in the first cell of the same row/column to form the figure in the third cell (Figure 2c);
- Intersection (IN): The third figure consists of only those elements of the figures that are displayed in both the first and the second cell (Figure 2d);
- Unique addition (UA): The third figure is made up only of those elements of the figures that are displayed in either the first or the second cell. Elements that are found in both figures are omitted (Figure 2e);
- Seriation (SE): The rule of change from the first to the second cell (e.g., movement, rotation, change in size, or addition of elements) is applied to the figure in the second cell to generate the figure for the third cell (Figure 2f);
- Variation of open Gestalts (VO): In each row/column, three one-dimensional figures (e.g., a line, curve, or arrow) are presented. These figures are then repeated in the other rows/columns, although not necessarily in the same order (Figure 2g). Open Gestalts in this case refers to the “one-dimensional” appearance (Hornke et al. 2000);
- Variation of closed Gestalts (VC): In each column/row, three different figures are presented (for instance, one square, one circle, and one pentagon; see Figure 2h). The order with which the three figures appear within their respective column or row is randomly determined. Closed Gestalts refer to figures of two-dimensional nature (Hornke et al. 2000), such as squares, rectangles, or other visually “closed” figures.

##### Item Construction

Items were generated using different operations and combining either 2 or 3 of those operations. We applied each of the 8 operations equally, often across the test, so that participants who were able to detect 1 specific operation particularly well would not disproportionally profit from this skill. Additionally, we applied each possible operation combination equally often for the same reason. Overall, 48 items were created. Each operation was included 15 times. As all items are balanced (i.e., each operation is included the same number of times) and we wanted to keep the combinations of operations equal, 24 items with 2 operations and 24 items with 3 operations were considered the best solutions to achieve balance within a mathematical (combinatoric) framework. The 24 two operation items were assigned to the first test half, and the 24 three operation items were assigned to the second test half.

##### Distractor Generation

Distractors were generated following the guidelines described in the introduction to make sure that participants could not dismiss certain distractors based on superficial features. All distractors were created so that they provided plausible solutions to the empty matrix cell. Plausibility was achieved on a structural level in that all but one distractor correctly featured at least one of the operations that define the attractor. Further, incorrect solutions to an operation were kept as closely related to the existing operations as possible. For example: In the example item in Figure 1, operation Seriation is represented by the black dot that rotates 90 degrees clockwise. The incorrect display of Seriation is depicted by either a black dot that is rotated 180 degrees or a black dot that is rotated 90 degrees counterclockwise. Visual similarity was achieved by using figural elements for the incorrect solutions that are the same or similar to the correct solution. For example: As shown above, Seriation is always featured by a black dot that is either at the correct position or at an incorrect position. The visual element, however, stays the same. In the same vein, the incorrect application of Addition is displayed by the correct figural element—a triangle—but either varied in size or orientation (see Figure 1). We further prevented counting as a means of achieving a correct response. In a final step, we constructed distractors so they could be evaluated to infer which operations participants applied/missed. To achieve these requisites, certain factors had to be considered:

##### Balanced Occurrence of Figural Elements

**Figure 3:**
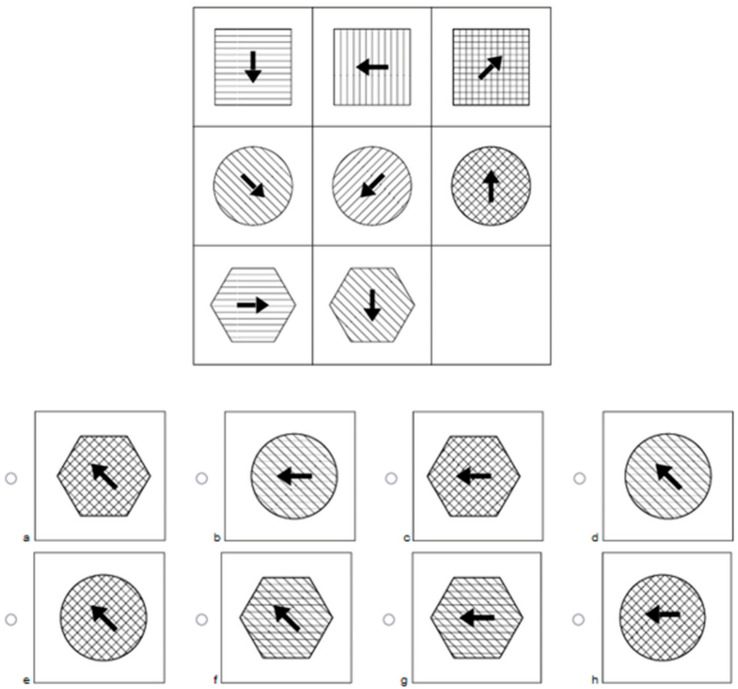
This example item features the three operations Identification, Addition, and Seriation. Response option f correctly follows all operations and hence completes the item stem.

In our test, all figural elements (e.g., arrows) appear equally, often across response options. As a result, participants could not estimate the likelihood of response options based on their appearance. For example, four response options might include an arrow pointing upwards, whereas the remaining four response options display an arrow pointing downwards. No response option could be ruled out heuristically. The same principle applies to the joint occurrence of figural elements (e.g., an arrow and the shape of the outer element). Some responses might show a circle with the arrow pointing upward, other responses a hexagon with the arrow pointing downward and vice versa. Thus, counting was not possible. An example is shown in Figure 3.

The item in Figure 3 features three operations: Identification, Addition, and Seriation. In this example, Identification refers to the outer shape of the figure: either a circle (false application of the operation) or a hexagon (correct application). Examining the eight response options, four options include a circle (b, d, e, and h), and four options include a hexagon (a, c, f, and g). Because both shapes occur equally often, they should be equally likely to be considered the correct shape for people who did not identify the Identification operation. The second operation in the example item is Addition. Addition in this item is displayed by the pattern inside the shapes. Again, four patterns resemble the correct application of this operation (b, d, f, and g), and four patterns represent a false application (a, c, e, and h). The last operation, Seriation, regards the direction of the arrows. There are four arrows pointing in the upper left-hand direction (a, d, e, and f; the correct display of the underlying operation) and four arrows pointing to the left-hand side (b, c, g, and h distractor elements of this operation). Again, each element is displayed the same number of times, and no element can be regarded as more or less likely. To conclude, it was not possible to rule out one of the elements by looking at the number of times each element appears. Furthermore, the combination these elements appear in also had to be considered.

Each combination of any two of the three figural elements was presented the same number of times. As can be seen in Figure 3, the two different shapes are both presented twice with the arrow pointing to the left and twice with the arrow pointing diagonally upwards, respectively, creating four different possible combinations that are all heuristically equally likely. This was to ensure that participants who correctly applied one operation were still left with response options that featured the same elements the same number of times. If this was not the case, counting would still be possible after successfully applying one operation. The technique of presenting each element and each combination of elements equally was often applied for every operation, resulting in eight unique response options. The combinations and thus the pattern of correctly applied operations can be presented in a table, as shown in Table 2.

##### Informative Content of Distractors

Several distractors were created so that they would serve as lures. In other words, they followed one of the operations necessary to solve the item, but not the other(s). The idea is that participants who selected these lures did so because they understood some part of the construction underlying the item, but not all of it. To be able to subsequently estimate which operations were correctly applied by a participant, a distractor for every possible combination of correctly/incorrectly applied operations was created. For example, distractors that displayed either the correct solution of operation A but not operation B, or operation B but not operation A, were included as well as distractors that did not display any correct features. For the three-operation items, this approach continued onto each possible two-way combination. For an item with operations ABC, apart from the attractor, there were three distractors where either one of the operations was correct (either A, B, or C), three distractors where any two-operation combination was correct (AB, AC, and BC, respectively), and one where none of the operations were correct. Figure 3 and Table 1 can also be used to exemplify this setup. The item in Figure 3 features three operations: Addition, Identification, and Seriation. The correct response is response option f.

Each distractor represents a different combination of a true/false depiction of the underlying operations. This is shown in Table 2. A participant selecting response option a could thus be assumed to have correctly detected and solved the operations Identification and Seriation, but not Addition. A participant selecting response option h, however, can be assumed to not have detected or solved any of the three operations. The emerging eight response options cover every combination of correct and incorrect application for each operation. Together with the guidelines presented above to include each element the same number of times, this distractor setup made it possible to identify operation knowledge for all operations while still preventing counting as an exclusion strategy.

#### 2.2.2. Berlin Intelligence Structure Test Short Form (BIS-S)

The short form of the Berlin Intelligence Structure Test (BIS-S) was used in sub-sample 4 (Beauducel and Kersting 2002; Jäger et al. 1997). The BIS-S consists of 15 tasks that capture the mental operations reasoning, creativity, memory, and speed in verbal, numerical, and figural contents. Overall, the BIS-S is a valid indicator for general intelligence (e.g., Beauducel and Kersting 2002). The BIS-S takes approximately 45–60 min to complete, depending on the time spent on the instructions and trial items for each task. An example task is the Charkow task: Participants of this task need to complete line drawings in accordance with certain rules. The first four drawings are given, and participants have to draw the remaining two. This task can be categorized into a figural reasoning facet of the BIS model (Jäger et al. 1997).

#### 2.2.3. Intelligence Structure Test 2000R (I-S-T 2000R)

The Intelligence Structure Test 2000R (I-S-T 2000R) was used as an additional measure of general intelligence in sub-sample 5 (Liepmann et al. 2001). With the I-S-T 2000R, three domains of cognitive abilities are tested: reasoning, knowledge, and memory. Reasoning and knowledge can each be divided into three subcategories: numeric, verbal, and figural. Furthermore, fluid intelligence (gf) and crystal intelligence (gc) are extracted from the reasoning and knowledge part of the test.

#### 2.2.4. Raven Advanced Progressive Matrices (RAPM)

The Raven Advanced Progressive Matrices (Raven et al. 1994) is a frequently used figural matrices test and was used as a benchmark for the newly developed test in one sub-sample. Each item presents a 3 × 3 matrix with one (the bottom right) empty field. Participants need to detect the underlying structure and decide which out of the eight given response options correctly continues the given matrix. Components can differ by form, color, size, or orientation and be applied horizontally or vertically. The RAPM consists of 48 items, with 12 items (Set I) being practice items. The actual test consists of 36 items (Set II).

#### 2.2.5. Need for Cognition (NFC) Scale

A German 4-item short form (Beißert et al. 2015) of the original NFC scale (Cacioppo and Petty 1982) was used in all sub-samples. The short version was used as it has been shown to have good psychometric properties and does not consume too much assessment session time (Beißert et al. 2015; Rammstedt et al. 2012). All 4 items (e.g., “I prefer my life to be filled with puzzles that I must solve”) of the scale employed a 7-point Likert scale that ranged from 1 (strongly disagree) to 7 (strongly agree).

### 2.3. Procedure

The test was always administered either in the laboratory or online via the online survey platform SoSci Survey (Leiner 2019). For participants who completed the test online from home, an online introduction was provided outlining the general aim of the study and testing procedure. Participants of sub-sample 4 who completed an additional battery of tests in the laboratory were introduced by an examiner. Examiners received training and clearly written instructions beforehand to guarantee objectivity in both test administration and test scoring when applicable. The introduction to the HeiQ (i.e., layout and number of items and response options, or introduction to the task) was embedded in the survey platform to ensure objectivity across all samples. Participants then had to complete two exemplary items (see Figure 1 for an example). The 5 sub-samples differed in the additional tests and surveys they had to complete. Samples 1–3 only completed the figural matrices test, the need for cognition questionnaire, and some basic demographic questions. No time limit was imposed. Participants took an average of *M* = 63.27 (*SD* = 36.44) minutes to complete the HeiQ. Sub-sample 4 additionally completed the BIS-S, and sub-sample 5 additionally completed the IST-2000-R and the RAPM. For these samples, participants first worked on the respective performance tests before receiving the HeiQ. They were then given a time limit of 60 min to complete the HeiQ. Participants took an average of *M* = 51.73 (*SD* = 10.00) minutes to complete the HeiQ.

## 3. Results

### 3.1. Percentage of Items Solved

Participants solved an average of *M* = 26.87 (*SD* = 10.70; Range = 3–47) out of the 48 items. Of the 24 2-operation items, *M* = 15.16 (63.17%); (*SD* = 5.26 (21.92%); and Range 3–24 (12.5–100%)) were solved. Of the 24 3-operation items, *M* = 11.74 (48.92%) and (*SD* = 6.10 (25.42%); Range 0–24 (0–100%)) were solved. Due to technical errors, 1 item was not correctly displayed for the first sub-sample, resulting in a possible maximum score of 47 for 155 people. Participants scored higher in the 2-operation condition (the first test half) than in the 3-operation condition (the second test half) *t* (681) = 22.38, *p* < .001, *d* = 0.86. The correlation between the 2 test-halves was *r* = 0.76 (*p* < .001). The correlation between the 2 test-halves corrected for attenuation was *r* = 0.87.

### 3.2. Missing Responses

The overall mean number of missing values was *M* = 2.53 (*SD* = 4.80) of 48 items. This included skipped responses (*M* = 0.36; *SD* = 1.33), overly fast responses (less than 5s per item; *M* = 1.03; *SD* = 3.17), and—for sub-sample 4—responses that could not be given because the time limit had been reached (*N* = 216; *M* = 2.83; and *SD* = 4.97). The correlation between the number of missing values and relative score (the percentage of correctly solved items out of the items that were not treated as missing) was *r* = −0.10 (*p* = .008). Taking only skipped and fast responses into account, participants missed *M* = 0.17 (*SD* = 0.70; range: 0–6) of 24 items in the first test half (2-operation items) and *M* = 1.23 (*SD* = 2.97; range: 0–22) of 24 items in the second test half (3-operation items). This difference was significant *t* (682) = −10.24, *p* < 0.001, *d* = −0.39. The number of skipped and fast responses correlated with a lower score on the remaining items at *r* = −0.26 (*p* < .001). Over all the sub-samples without a time limit (*N* = 388), performance on the first test half predicted the number of missing values in the second test half, *R*^2^ = 0.06 (adjusted *R*^2^ = 0.06), *F* (1, 386) = 25.89, *p* < .001. The correlation of the number of items that were not reached due to the time limit and the relative score on the valid items was *r* = 0.11 (*p* = .004). Percentages of participants who correctly solved the item as well as the number of valid responses are presented in Table 3.

### 3.3. Measurement Models

We used a Rasch model (Rasch 1960) to analyze the data. A Rasch dichotomous model is the most parsimonious model within probabilistic test theory and is sometimes also called a 1PL model. The Rasch model specifies that the probability of a person (v) for solving item (i) is a function of the ability of this person (theta $\theta_{v}$) and the difficulty of this item $\sigma_{i}$. The higher the ability $\theta_{v}$ and/or the lower the difficulty $\sigma_{i}$, the higher the probability of solving the item correctly $Ρ\left(\chi_{\nu i}=1\right)$. In mathematical terms, the equation is rendered as follows:

(1) $$Ρ\left(\chi_{\nu i}=1\right)=\frac{e^{\left(\theta_{\upsilon }-\sigma_{i}\right)}}{1+e^{\left(\theta_{\upsilon }-\sigma_{i}\right)}}$$

As there are only two parameters in a Rasch model, item discrimination is uniform. This means that every item loads equally on the same latent construct. Although item responses are not missing at random (MAR), item and person parameter estimates of an IRT measurement model are robust and can be incorporated as such (Pohl et al. 2013). Measurement models were estimated using the means and variance adjusted weighted least square estimator (WLSMV) implemented in Mplus 8.6 (Muthén and Muthén 2017). Model fit indices are reported in Table 4. We further report outfit, infit, differential item functioning, and item difficulties for all items in Table A1 in the Appendix A.

All models were accepted according to the standardly used fit indices: With a RMSEA of ≤0.06, the Rasch model showed a good model fit (Hu and Bentler 1999; Yu 2002). An *χ*^2^*/df* ratio ≤ 5 also suggests an acceptable model fit (Wheaton et al. 1977).

### 3.4. Reliability

The split-half reliability coefficient, computed as an odd–even split, was *r* = 0.88 *p* < .001 (Spearman–Brown corrected). As a measure of internal consistency, Cronbach’s α and Revelle’s omega total (see McNeish 2018) were computed for the overall test and for the operation indicators. As there were many participants with missing data and data are assumed not to be missing at random (NMAR) (Pohl et al. 2013), listwise deletion would lead to an overestimation of alpha. As a result, pairwise deletion was imposed. With a Cronbach’s α = 0.93 and a Revelle’s omega total ω_RT_ = 0.94, the overall test had excellent internal consistency according to the common conventions. We further computed the marginal empirical reliability based on the squared standard errors of the sample scores using the expected a posteriori (EAP) estimator as suggested by Brown and Croudace (2015). Marginal empirical reliability was ρ = 0.91. A subsample of *N* = 204 took the test twice. On average, the test–retest interval for this sub-sample was *M* = 87 days (*SD* = 19). The retest-correlation was *r* = 0.88 *p* < .001 (Spearman–Brown corrected).

### 3.5. Validity

The HeiQ’s validity was assessed by investigating its correlation with a variety of other intelligence measures, academic performance indicators, and other related constructs. In sub-sample 5 (*N* = 76), the HeiQ scores were highly correlated with the RAPM *r* = 0.81 *p* < .001. The correlation corrected for attenuation was *r* = 0.90. In the same sub-sample, the correlation of the HeiQ with the I-S-T 2000R’s general intelligence scale was *r* = 0.73 *p* < .001, and its correlation with the I-S-T 2000R’s general knowledge scale was *r* = 0.42 *p* < .001, speaking to the test’s convergent and discriminant construct-related validity. The correlational pattern of the HeiQ with the IST’s sub-scales is displayed in Table 5. In sub-sample 4 with *N* = 215 valid BIS-s scores, the correlation between the BIS-s and the HeiQ was found to be *r* = 0.58 *p* < .001. An overview of the association of the HeiQ with other intelligence test measures can be found in Table 5. We further report the descriptive statistics and correlations of all measures in Table A2 in the Appendix A. All measures were scored according to the advised scoring procedure in the respective test manuals. Namely, every correct response was scored as one, whilst every incorrect response was scored with zero. Sum scores for the scales of the BIS-S and the I-S-T 2000R were then calculated. The IQ score of the BIS-S was drawn from the tables included in the manuals. For the other measures, the respective sum scores were used.

Table 6 shows the correlations of the HeiQ with academic achievement indicators. Only participants with a German high-school qualification (Abitur) noted as GPA were included to guarantee comparability. In the German school system, a lower grade indicates better performance; thus, grades are expected to be negatively correlated with reasoning abilities. HeiQ scores and GPA were indeed negatively correlated; *r* = −0.38 *p* < .001. Similarly to Heydasch (2014), we also calculated correlations between GPA and HeiQ scores for participants under 24 only. Inclusion of a large age range can skew GPA measures, as the school and grading system underlies changes over time.

For participants under the age of 24, the HeiQ and GPA were strongly correlated; *r* = −0.48 *p* < .001. Furthermore, grades for the 5 different academic subjects were provided by the participants of sub-sample 4 (i.e., Mathematics, German, English, Biology, and Arts). As these grades are provided in terms of achievement points (ranging from the worst of 0 to the best of 15 points), grades should be positively correlated with the HeiQ. The HeiQ scores correlated highest with grades in mathematics *r* = 0.48 *p* < .001 (*r* = 0.48 *p* < .001 for participants under 24) and biology *r* = 0.32 *p* < .001 (*r* = 0.44 *p* < .001 for participants under 24). Lower correlations with German, *r* = 0.20 *p* < .01 (*r* = 0.22 *p* < .05 for participants under 24) and English, *r* = 0.17 *p* < .05 (*r* = 0.20 *p* < .05 for participants under 24) were found. The grade in Arts was not significantly associated with the HeiQ; *r* = 0.08, *p* = .43 (*r* = 0.17 *p* = .18 for participants under 24).

As sub-sample 5 completed both the RAPM and the HeiQ, separate correlations for GPA were computed. The correlation between GPA and the HeiQ score for those participants of sub-sample 5 that held a high school diploma (*N* = 72) was *r* = −0.49 *p* < .001. The correlation between GPA and the RAPM was *r* = −0.38 *p* = .001. The difference of *r* = −0.11 was significant (*Fisher’s z* = 1.67, *p* = 0.048) (Meng et al. 1992).

### 3.6. Operation-Specific Indicators

Additional scores for each cognitive operation for every participant were computed. Hereby, each response given by a participant was analyzed for the operations that were included to construct the item. For each correctly identified operation, participants received a point on that operation’s scale. For example, if a respondent completed the item shown in Figure 3 and chose response option b, d, f, or g, they correctly applied the operation Addition and, accordingly, would receive one point on the Addition scale. This was done for each participant and operation across all items. As each operation occurred on 15 test items, the resulting sum scores could vary between 0 and 15. The mean percentages of correctly identified operations are presented in Table 7.

A linear regression analysis was run to test if the HeiQ scores predicted GPA. Similarly to the analyses above, 2 separate regressions were run for the whole sample and for participants under the age of 24. The HeiQ total score significantly predicted high school GPA *F* (1, 470) = 78.94, *p* < .001. Further, *R*^2^ for the overall model was *R*^2^ = 0.14 (adjusted *R*^2^ = 0.14). For people under 24, *R*^2^ for the overall model was *R*^2^ = .23 (adjusted *R*^2^ = 0.23). This prediction was also significant *F* (1, 262) = 78.45, *p* < .001. A multiple regression was computed to test whether the operation-specific indicators led to an increase in an explained variance of GPA compared to the regression analysis for the overall test score. For people under 24, *R*^2^ increased from *R*^2^ = .23 to *R*^2^ = 0.29 (adjusted *R*^2^ = 0.27) (*F* (9, 254) = 11.67, *p* < .001). For the whole sample, *R*^2^ increased from *R*^2^ = 0.14 to *R*^2^ = 0.18 (adjusted *R*^2^ = 0.17) (*F* (9, 462) = 11.44, *p* < .001).

## 4. Discussion

In this study, we introduced a novel figural matrices test, the HeiQ. The test was developed to overcome construction-related shortcomings in previous matrices tests, or more specifically, to reduce the use of bypassing strategies to increase control of item difficulties, and by effect, to improve reliability and validity. These aims were achieved by adopting a systematic distractor construction approach (see Guttman and Schlesinger 1967). Because distractors were generated in accordance with a facet design, items of the HeiQ cannot be solved by counting the properties of response options. Because every correct or incorrect feature of an operation is presented equally often, none of the distractors can be eliminated without correctly applying at least one of the item’s underlying operations. Additionally, distractors were kept as visually similar to the attractor as possible, meaning that distractors and the attractor featured similar figural elements (if the correct application of an operation consisted of a circle, the incorrect responses also featured a circle, but differed, for example, in size or position). Finally, distractors were constructed so that they appeared to be plausible solutions to the item. All but one distractor features a correct application of at least one of the operations, thus guaranteeing structural similarity between distractors and the attractor. Taken together, these measures were designed to minimize the use of response elimination strategies.

The application of a systematic distractor construction approach was successful, as the HeiQ showed very good psychometric properties. The test proved to be Rasch scalable, which supports the unidimensionality of the test. This is in line with the previous research on the dimensionality of figural matrices tests (Arthur and Woehr 1993; Waschl et al. 2016). In the measurement model, factor loadings of all items could be fixed to one, meaning that all items weighed the same and suggesting that no corrections are necessary when items are combined. Consequently, the test enables the calculation of a sum score of the solved items and use of these sum scores to compare participants regarding their reasoning abilities. We have further tested a Birnbaum (2PL) model, where the probability for a person to solve an item is dependent on the ability of the person, the difficulty of the item, and an additional discrimination parameter of the item. This model fit the data better than the Rasch model. However, as the Rasch model still achieved an acceptable fit, according to conventional cut-off criteria, we preferred the more parsimonious model for our study. Furthermore, there was considerable test score variance in all sub-samples, and items of varying difficulty discriminated well between participants within all sub-samples. Both the two-operation as well as the three-operation items performed well in our test, and manipulating the number as well as the kind of operations used to construct an item seemed successful in creating items of varying difficulty. Controlling for bypassing strategies might have further enhanced the difficulty distribution of the generally easier two-operation items. Despite the fact that we collected general population and student-only sub-samples, there was no evidence of floor or ceiling effects in any sub-sample. We therefore concluded that HeiQ is a suitable tool for the assessment of reasoning abilities across different academic levels.

A high Cronbach’s alpha, Revelle’s omega total, marginal empirical reliability, and a high split-half reliability coefficient were observed for the HeiQ. The high retest-correlation speaks to the test scores’ stability over time. In sum, there is good evidence that the test scores obtained with the HeiQ are very reliable.

The HeiQ test scores correlated strongly with other intelligence measures, such as the RAPM, the BIS-S, and the I-S-T 2000R. The correlations in the different sub-samples were all between 0.58 and 0.81 in size, which is comparable with the results obtained in similar validation studies of previous figural matrices tests, for example, the HMT by Heydasch (2014). The correlations after correcting for attenuation were even higher, indicating that the HeiQ’s construct validity is excellent.

HeiQ test scores were strongly correlated with the GPA of all participants (*r* = −0.38) and even stronger with those of participants under the age of 24 (*r* = −0.48). These correlations were substantially higher than those observed with the HTM, for example (*r* = 0.19 and *r* = 0.34; see Heydasch (2014)). Furthermore, for sub-sample five that completed both the RAPM and the HeiQ, the correlation between the HeiQ and GPA was significantly higher than the correlation between the RAPM and GPA. These findings suggest that the HeiQ’s criterion-related validity is also exceptionally high.

A further advantage of our new test is that incorrect solutions can be used to assess which operations were correctly applied during the attempt to solve the item. When using operation-level scoring, the criterion-related validity of the HeiQ could be improved even further. An investigation of reasoning abilities may also be useful in order to gain better insight into the processes underlying reasoning abilities. We will elaborate on this point below as an avenue for future research.

### 4.1. Considerations on Bypassing Strategies Other Than Response Elimination

Although we tried to account for guessing-without-attempting-to-solve by treating overly fast responses as missing, it is not clear whether participants diligently worked on each item. This has been shown to threaten the validity of performance tests (Wise and DeMars 2010). If participants are not motivated to perform well on a test, they will either randomly choose one response option without applying any solution strategy or correctly apply one operation and then choose between the response options which feature this operation. In the latter case, participants would switch to simpler decision heuristics that they might still consider to be “good-enough”. In line with this strategy, Gonthier and Roulin (2020) pointed out that when solving matrices tests, participants apply a cost–benefit trade-off similar to satisficing in complex problem-solving heuristics (Gonthier and Roulin 2020). These authors further showed that, independent of their abilities, motivated participants will try to actively solve the matrices for a longer period than unmotivated participants, resulting in a higher test score than their peers of similar or even higher ability. The consequence of the simpler decision heuristic is that participants might sometimes attain a correct response even though they have only solved two out of three operations. This is due to the smaller number of distractors, that—in that case—include the correct features of the two operations that were solved. We are aware that this is in some way problematic, but believe there is no way to inhibit points achieved by guessing altogether. The only test type which is able to counter any kind of heuristic solution strategy is a distractor-free version of a figural matrices test introduced by Becker et al. (2014). In this computerized test, participants generate their own response by selecting figural elements from pre-existing lines and shapes and dragging them into the response field. However, distractor-free matrices tests also have their downsides. The most serious problems with these tests compared to standard multiple-choice tests are as follows: They are more difficult to implement as they require additional instructions and practice, they take more time to be solved, and there is a high variability among participants regarding how much time it takes them to solve the test, and they are more difficult to score (automatic scoring is not possible). For these reasons, we believe that the use of distractors is preferable. Further, we see that our distractor design keeps points achieved by guessing over 48 items to a minimum.

### 4.2. Future Applications and Opportunities of the Operation-Level Test Scoring

It has been argued that response tendencies in matrices tests can be used to identify thought processes (Laurence and Macedo 2022). In the results section, we made use of the HeiQ’s systematic distractor design to calculate test scores that supposedly reflect operation-level knowledge. These scores reflect the proportion of correctly detected and applied operations, namely, how many times a participant applied a particular operation correctly out of the total number of items that included this operation. Furthermore, an overall operation score could be computed, where every correctly identified operation is awarded one point, instead of merely scoring the complete item as being either correct or incorrect. Such a scoring procedure also takes into account how many operations were identified correctly when attending an item (i.e., zero, one, two, or three). Test scores achieved with this scoring procedure correlated more strongly with GPA than scores obtained with the simple correct/incorrect scoring. This finding suggests that these scores contain information over and above the average solution scores. The operations used in the HeiQ have shown to be of varying difficulty for the participants, which can be inferred from the large standard deviations.

One possible route for future research could be to investigate cognitive training effects in competence testing. Research has shown that, through repeated test taking, the total score of the test increases (Bors and Vigneau 2003). Bors and Vigneau (2003) have pointed out that this improvement was highly variable and not just due to participants answering more items compared to the first testing session. They further found that improvement was not due to memory effects of specific items, as participants frequently switched from the correct to incorrect response choice and vice versa. They concluded that learning relates to how items are solved and to not remembering certain items. All of this information cannot be captured by a total sum score that merely shows the number of correctly solved items. Thus, to identify determinants of cognitive performance, one needs to shift from an aggregate score to a more detailed analysis of individual differences (Bors and Vigneau 2003), and our newly developed test with the possibility of focusing on operation level changes may be a good tool for doing so.

With the HeiQ, both a change to the aggregate score and a breakdown of training effects to the individual operation level were observed and may thus be attributed to individual ability, strategies, or handling of certain operations. Researchers can also use this knowledge to analyze whether specific operations show higher learning curves than others. As all operations are included equally often, in-test training effects are assumed to be constant for each operation. Because the figural matrices test does not use verbal tasks, it may be useful for assessing reasoning within participants with restricted verbal abilities (Carpenter et al. 1990).

## 5. Conclusions

The HeiQ demonstrated excellent reliability as well as construct- and criterion-related validity estimates. The test successfully limits counting, and no distractor can be excluded without at least one operation being applied correctly. The underlying distractor design further encourages participants to use constructive matching as compared to bypassing strategies that are correlated with lower validity. Another excellent advantage of the current test is the possibility of forming operation-specific indicators based on a rule-based distractor analysis. Attaining a more detailed score than a sum score can aid in a more precise ability to estimate participants and diagnose their differences. The operation-specific indicators retrieved from the HeiQ showed good psychometric properties and explained more variance in external criteria than the sum scores.

## Figures and Tables

**Table 1:** Sub-sample specific demographics and assessed measures.

Sub-Sample	*N*	Age		Gender (Female (%))	Population	Location	Cognitive Measures	Additional Questionnaires	Academic
		*M*	*SD*						
1	155	23.81	5.49	112 (72.3)	University	Online			
2	107	24.18	3.42	72 (67.3)	University of Applied Sciences	Online		NFC	GPA
3	126	24.66	4.47	92 (73.0)	University of Applied Sciences	Online		NFC	GPA
4	216	26.02	11.18	136 (63.0)	University and general population	In person	BIS-S	NFC	GPA
5	79	33.81	13.54	40 (50.6)	General population	Online	I-S-T 2000R RAPM	NFC	GPA

Note: NFC: Need for Cognition. GPA: Grade point average for high school and university degree (if applicable). BIS-S = Berlin Intelligence Structure Test Short Form. I-S-T 2000R = Intelligence Structure Test 2000R. RAPM = Raven Advanced Progressive Matrices.

**Table 2:** Distractors and their correct/incorrect setup of underlying operations for the example item.

Response Option	Addition	Identification	Seriation	Operations Correct
A	0	1	1	2
B	1	0	0	1
C	0	1	0	1
D	1	0	1	2
E	0	0	1	1
F	1	1	1	3
G	1	1	0	2
H	0	0	0	0

Note: Response option labels refer to the letter assigned in Figure 3. The number 1 is used to specify that the operation is correctly displayed in the response option, the number 0 when the operation is incorrectly displayed. Response option f is the correct response; hence, all three operations are correctly displayed.

**Table 3:** Percentage correctly solved and missing responses per item.

Item	Correctly Solved (in %)	Valid Responses (*N*)
1	50.66	677
2	60.59	680
3	51.19	670
4	66.03	680
5	43.91	681
6	93.27	683
7	41.38	679
8	48.32	683
9	70.90	677
10	89.00	682
11	27.71	682
12	34.85	680
13	69.96	679
14	81.11	683
15	60.35	681
16	60.03	683
17	71.98	678
18	79.18	682
19	80.32	681
20	81.47	680
21	71.76	680
22	55.67	670
23	86.78	681
24	48.07	672
25	58,49	677
26	73.63	675
27	66.17	677
28	36.61	672
29	71.58	665
30	23.72	662
31	51.51	662
32	36.67	660
33	76.28	666
34	62.11	665
35	60.76	660
36	59.51	657
37	44.73	626
38	46.63	489
39	51.81	635
40	49.03	620
41	38.21	602
42	40.27	596
43	66.39	607
44	42.37	557
45	55.06	563
46	55.50	564
47	55.12	557
48	55.29	539

Note: Percentage correctly solved refers to the people who attempted the item (excluding missing responses).

**Table 4:** Model fit indices for Rasch measurement model.

Model	*χ* ^2^	*df*	*p*	CFI	RMSEA	*χ*^2^/*df*
	3542.68	1127	<.001	0.88	0.056	3.14

Note: CFI = comparative fit index; RMSEA = root mean square error of approximation; *N* = 683.

**Table 5:** Correlations between the HeiQ and intelligence measures.

Variable		Sample Size (*N*)	Correlation
RAPM		76	0.81 *** (0.90)
BIS-S		215	0.58 *** (0.70)
I-S-T 2000R		76	
	Reasoning		
	Overall		0.73 *** (0.79)
	Verbal		0.42 *** (0.49)
	Numeric		0.66 *** (0.71)
	Figural		0.63 *** (0.79)
	Knowledge		
	Overall		0.43 ** (0.47)
	Verbal		0.23 (0.27)
	Numeric		0.51 *** (0.61)
	Figural		0.39 *** (0.48)

Note: RAPM = Raven Advanced Progressive Matrices; BIS-S = Berlin Intelligence Structure Test Short Form; I-S-T 200R = Intelligence Structure Test 2000R. The numbers in parentheses refer to the correlations corrected for attenuation. ** *p* < .01, *** *p* < .001.

**Table 6:** Correlations between HeiQ scores and academic achievement indicators.

Variable		Sample Size	Correlation
High School			
	GPA	472 (264)	−0.38 *** (−0.48 ***)
	Mathematics	194 (126)	0.48 *** (48 ***)
	German	192 (125)	0.20 ** (0.22 *)
	English	186 (118)	0.17 * (0.20 *)
	Biology	142 (91)	0.32 ***(0.44 ***)
	Arts	107 (64)	0.08 (0.17)

Note: Numbers in parentheses refer to participants under the age of 24. * *p* < .05, ** *p* < .01, *** *p* < .001.

**Table 7:** Mean, standard deviation, Cronbach’s alpha, and correlations among operation indicators.

Operation	*M*	*SD*	Alpha	1	2	3	4	5	6	7	HeiQ	BIS
Addition	81.69	15.23	0.68								0.74 *** (0.93)	0.38 *** (0.51)
Subtraction	77.86	19.51	0.77	0.67							0.83 *** (0.98)	0.55 *** (0.70)
Identification	91.93	11.08	0.66	0.61	0.62						0.55 *** (0.70)	0.32 *** (0.44)
Variation of Open Gestalts	77.95	16.38	0.64	0.63	0.64	0.61					0.72 *** (0.93)	0.42 *** (0.58)
Variation of Closed Gestalts	78.05	15.98	0.62	0.62	0.63	0.56	0.65				0.72 *** (0.95)	0.40 *** (0.56)
Intersection	63.44	20.21	0.69	0.58	0.65	0.47	0.56	0.54			0.81 *** (0.99)	0.42 *** (0.56)
Unique Addition	61.79	24.44	0.80	0.61	0.72	0.50	0.59	0.56	0.72		0.84 *** (0.97)	0.51 *** (0.63)
Seriation	76.60	19.16	0.73	0.65	0.72	0.57	0.64	0.62	0.66	0.69	0.80 *** (0.97)	0.53 *** (0.69)

Note: Variation of Closed Gestalts, Unique Addition, and Seriation only consist of 14 items for *N* = 155 people, since 1 item was erroneous and had been excluded from analysis. The numbers in parentheses refer to the correlations corrected for attenuation. *** *p* < .001.

## Data Availability

The data reported in this article can be requested from the authors.

## Appendix A

**Table A1:** Outfit, infit, differential item functioning, and item difficulties for all items.

Item	Outfit Mean-Square	Infit Mean-Square	Differential Item Functioning	Item Difficulty
1	1.513	1.367	1.976 *	−0.027
2	0.989	1.014	1.554	−0.430
3	1.020	0.985	−1.068	−0.048
4	0.695	0.841	−0.305	−0.662
5	1.521	1.316	0.373	0.246
6	0.737	0.859	0.384	−2.395
7	1.176	1.117	0.755	0.349
8	1.167	1.148	0.602	0.068
9	1.361	1.193	0.397	−0.882
10	0.949	0.959	1.000	−1.964
11	0.960	0.913	−1.458	0.947
12	1.065	1.050	−1.663	0.623
13	0.802	0.856	−0.939	−0.838
14	1.062	1.068	−0.935	−1.413
15	0.914	0.903	−2.190 *	−0.420
16	0.839	0.924	−0.701	−0.407
17	0.749	0.807	−0.344	−0.932
18	1.227	1.194	0.432	−1.301
19	1.311	1.019	0.455	−1.366
20	0.590	0.796	−0.464	−1.434
21	1.034	0.934	−1.796	−0.922
22	0.815	0.855	−0.821	−0.228
23	0.821	0.882	−1.105	−1.788
24	0.672	0.752	−2.559 *	0.078
25	1.117	1.094	2.307 *	−0.344
26	0.983	0.932	−1.878	−1.012
27	0.851	0.930	−1.360	−0.668
28	0.895	0.937	−0.848	0.548
29	0.868	0.895	−0.673	−0.913
30	1.437	1.215	0.122	1.146
31	1.235	1.141	−0.239	−0.061
32	0.885	0.895	−1.836	0.546
33	0.978	1.005	1.361	−1.145
34	1.058	1.007	−1.102	−0.494
35	1.666	1.396	6.102 *	−0.437
36	0.809	0.877	−0.881	−0.386
37	0.846	0.890	−0.335	0.212
38	1.435	1.330	−0.118	0.136
39	0.783	0.837	−1.425	−0.073
40	0.780	0.815	−0.137	0.039
41	0.697	0.765	−0.968	0.481
42	1.109	0.998	0.967	0.395
43	0.977	1.037	1.196	−0.678
44	0.765	0.802	0.826	0.308
45	0.936	0.986	2.588 *	−0.204
46	0.856	0.896	2.396 *	−0.221
47	0.970	0.975	1.006	−0.206
48	0.957	0.966	2.155 *	−0.213

Note: * = *p* < .05.

**Table A2:** Descriptive statistics and correlations of all measures.

Measure	*M*	*SD*	Range	Skewness	Curtosis	1	2	3.1	3.2
HeiQ	26.87	10.68	3–47	0.04	−1.03				
BIS-S	97.63	18.23	55–145	−0.27	−0.30	0.58 *** (0.70)			
I-S-T 2000R									
Reasoning	113.53	21.89	62–153	−0.18	−0.64	0.73 *** (0.79)	-		
Knowledge	54.72	11.76	21–78	−0.42	−0.09	0.43 ** (0.47)	-	0.53 *** (0.58)	
RAPM	23.64	6.58	9–35	−0.34	−0.90	0.81 *** (0.90)	-	0.73 *** (0.81)	0.36 ** (0.41)

Note: The correlations with the HeiQ differ in sample size, as they refer to different sub-samples. They are as follows: HeiQ and BIS-S *N* = 215; HeiQ and I-S-T 2000R and RAPM *N* = 76. Descriptions of the HeiQ are given based on the whole sample. As no participant took the I-S-T 2000R and the BIS/RAPM, there are no correlations for this field. The numbers in brackets refer to the correlations corrected for attenuation. ** *p* < 0.01, *** *p* < 0.001
